# Resistance to HIV Integrase Inhibitors: About R263K and E157Q Mutations

**DOI:** 10.3390/v10010041

**Published:** 2018-01-18

**Authors:** Charlotte Charpentier, Diane Descamps

**Affiliations:** IAME, UMR 1137, INSERM, Université Paris Diderot, Sorbonne Paris Cité, AP-HP, Laboratoire de Virologie, Hôpital Bichat, AP-HP, 75018 Paris, France; diane.descamps@aphp.fr

**Keywords:** HIV, integrase, R263K, E157Q

## Abstract

The use of integrase inhibitors (INI) is increasing in antiretroviral therapies (ART) and INI are not all equal regarding genetic barrier to resistance. The aim of this manuscript was to review main in vivo and in vitro knowledge about two particular integrase resistance-associated mutations: R263K and E157Q. The R263K mutation was the first mutation rarely found selected at time of virological failure in patients failing a first-line dolutegravir-based treatment. Further in vitro studies on R263K mutants showed a moderate increase in phenotypic resistance level and a drastic reduction in viral replicative capacity. No compensatory mutations were evidenced. The E157Q mutation is polymorphic, found between 1.7% and 5.6% of viral sequences issued from ART-naïve patients depending on the viral subtype; as well as acquired resistance emerging at failure of a raltegravir-based regimen in two case reports. We reported data on phenotypic resistance level of E157Q mutants and virological response of patients harboring a E157Q virus initiating an INI-based regimen, showing that dolutegravir might be the most recommended INI in such patients. These findings show that there is still a need for a better understanding of resistance mechanisms to INI and emphasized the importance of genotypic background in viral evolution under drug pressure.

## 1. Introduction

Human Immunodeficiency Virus (HIV) integrase inhibitors (INI) are the most common drug class recommended by the different international guidelines as third agent of the combined antiretroviral therapy (cART). First licensed INI, raltegravir (RAL), is now available for 10 years and determinants of genotypic resistance are well-described for this drug, as well as for the second first-generation INI, elvitegravir (EVG). However, there is still data to provide regarding resistance mechanisms to the most recent, second-generation INI, dolutegravir (DTG).

In this manuscript, we have reviewed main in vivo and in vitro knowledge about two integrase resistance-associated mutations: R263K and E157Q. R263K mutation is interesting because this mutation was selected in vivo at failure of a DTG-based regimen and only displayed a moderate increase in phenotypic resistance level [1,2]. Furthermore, Mark Wainberg and his team conducted several studies regarding in vitro characteristics of R263K integrase mutants. E157Q integrase mutation is of interest too, since it is both polymorphic, with variable prevalence in INI-naïve patients depending on the viral subtype [3], but also selected at virological failure (VF) of a RAL-based regimen in two case reports [4,5] and described in a case report of a non virological response to a DTG-based regimen [6].

## 2. R263K Integrase Mutation

### 2.1. First In Vitro Data on R263K Mutant

In the first report of in vitro selection under DTG pressure conducted in primary human cells, the R263K substitution in integrase was identified through culture selection after 20 weeks as a DTG resistance-associated mutation [2]. Furthermore, in these selection experiments, S153Y and S153T substitutions were observed in combination with R263K in one subtype B and in one subtype C virus, respectively [2]. In the study of Quashie et al., site-directed mutagenesis analysis showed that R263K did confer very low-level resistance to DTG (Fold Change (FC) = 1.06), a slight increased for EVG (FC = 1.75) and no change for RAL (FC = 0.63) [2]. More recently, it has been shown that in both single and multiple rounds of HIV-1 infections, bictegravir (BIC) and cabotegravir (CAB), two INIs currently under development, remained active against R263K mutant [7].

To date, in vitro selection experiments failed to select viruses with a high level of resistance to DTG, except when very high concentrations of DTG, until 500 nM, were used. Interestingly, no integrase resistance-associated mutations were detected in this resistant selected virus. However, this work of Malet et al. reported, for the first time, an INI-resistant virus with mutations selected outside integrase gene, located in the 3’ PPT region [8].

### 2.2. First In Vivo Data on R263K Mutation Selection

In the SAILING randomized trial, which included cART-experienced but INI-naïve patients, selection of INI resistance at time of VF through week 24 was observed in two participants among the DTG arm (*n* = 354 patients). In both cases the R263K mutation was selected with a plasma viral load at failure comprised between 3 and 4 log_10_ c/mL [1]. One virus displayed R263K as a single mutation and phenotypic analysis of this clinical isolate showed a FC to DTG and RAL of 1.12 and 0.96, respectively, with a reduced viral replicative capacity equal to 33% [9]. The second virus harbored the V260I mutation added to the R263K, this double-mutant resulted in a FC to DTG and RAL of 1.93 and 1.12, respectively [9]. The single mutant V260I did not confer DTG or RAL FC increase [9]. In addition, R263K site-directed mutant analyses using MT4 cells in a 5-day assay with cell tier glow readout showed a FC of 2.1, 0.8 and 10.6 for DTG, RAL and EVG, respectively [9]. A few additional VF occurred in the SAILING trial after W48 and R263K mutation was detected in one of them [10]. In this latter, VF occurred at week 120 with a viral load of 622 c/mL and R263K was detected added to A49G and S230R integrase mutations. This accumulation of integrase mutations probably resulted from the long duration of replication under treatment, since plasma viral load was above 50 c/mL since week 96. This triple-mutant clinical isolate showed an increased DTG FC of 5.77 and a RAL FC of 2.62, with a very low viral replicative capacity of 12% [10].

### 2.3. Prevalence of R263K among cART-Naïve Patients

Regarding its prevalence, R263K mutation is very rare in cART-naïve patients, in the French epidemiological transmitted drug resistance survey conducted in patients in primary infection with a prevalence of 0.9% (*n* = 2/233 patients) [11]. In a study based on 92 recently diagnosed, but chronically-infected, cART-naïve patients, no R263K was detected by Sanger sequencing technology and was found in two samples in minority proportion only when using ultra-deep sequencing technology [12].

### 2.4. In Vitro Characterization of R263K Mutants

**Figure 1:**
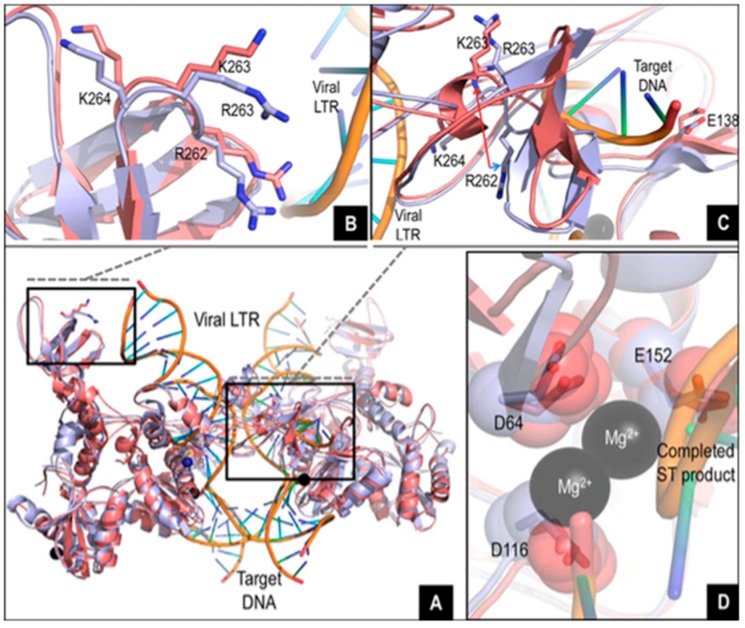
In silico studies of the wild-type and R263K integrases (**A**–**D**) adapted from Figure 4 of reference [2]. Overlay of the wild-type and R263K integrases, intasome and strand transfer complex models with viral LTR DNA and target DNA. The tetrameric IN structure is composed of the inner and outer subunits; (**B**) Detailed view (8 Å) of the overlay showing proximity between residue 263 in one of the outer subunits and the viral LTR; (**C**) Detailed view (12 Å) showing the pronounced shift in localization and orientation of residue R262 in the presence of the R263K mutation at the vicinity of the target DNA in one of the inner subunits; (**D**) Close-up overlay showing the relative positions of the D_64_D_116_E_152_ core catalytic residues in the wild-type and R263K enzymes in the inner subunits.

The study of Quashie et al. showed that the presence of R263K mutation did confer a decreased integration in cell culture without altering reverse transcription step [2]. Further in vitro experiments performed in this study, including biochemical cell-free assays performed with purified integrase enzyme containing R263K mutation, showed a slight decrease in 3′processing and strand transfer activities compared to the wild-type virus. Structural modeling suggested that the R263K mutation affects integrase-DNA interactions and in vitro integrase-DNA binding assays confirmed these data [2] (Figure 1). In the study of Mesplède et al., they performed prolonged infections by transferring culture fluids from infected cells to uninfected cells at weekly intervals and it resulted in a progressive decrease in integrated viral DNA between weeks 2 to 4 of infection. Thus, prolonged infections with R263K mutants led to a progressive decline in integrated HIV-1 DNA over time [13].

Recently, the study of Kessl et al. showed that IN binding to the viral RNA genome is necessary for formation of infectious viral particles. Interestingly, the residues identified in IN to mediate IN-RNA interactions are residues 269–273, very close to the 263 residue [14]. This could participate to the drastic reduction of the viral replicative capacity observed in case of the presence of the R263K mutation.

### 2.5. R263K and Potential Compensatory Mutations

It has been shown that R263K mutants display a low viral replicative capacity [2]. Thus, several studies were conducted to identify potential secondary mutations that would restore viral replicative capacity. First studies reported on the M50I integrase polymorphism, since it was selected in culture secondary to the R263K mutation [2]. The findings showed that M50I polymorphism in combination with R263K increased resistance to DTG in tissue culture and in biochemical assays but did not restore viral replicative capacity of the R263K mutant [15]. Similarly, H51Y integrase mutation also emerged secondary to R263K mutation in DTG selection experiments. So, in vitro characteristics of H51Y single- and H51Y-R263K double-mutants were studied showing that the addition of H51Y to R263K increased phenotypic resistance level to DTG with a FC of 16.5, whereas H51Y alone did not confer resistance to this drug. However, the addition of H51Y is accompanied by dramatic decreases in both enzymatic activity and viral replication. So, the H51Y mutation is also not a compensatory mutation to the R263K [16].

The study of Liang et al. showed that the T66I substitution emerged from a wild-type virus but also from a R263K mutant and from a E138K-R263K double-mutant virus under RAL or EVG pressure [17]. The aim of this study was to assess the effects of the T66I and E138K substitutions, alone and in combination with R263K, on viral replicative capacity and resistance to INI. They showed that the addition of R263K to the T66I substitution did not significantly compromise susceptibility to DTG, while decreasing both viral replicative capacity and strand-transfer activity [17]. The addition of the E138K substitution to the T66I-R263K double-mutant partially compensated for these deficits and resulted in a high level of resistance against EVG but not against DTG or RAL [17].

Interestingly, E157Q mutation is able to partially restore decrease in integrase enzymatic activity caused by the R263K substitution, thereby acting as a possible secondary, compensatory mutation. Furthermore, the E157Q-R263K double-mutant also displayed enhanced DTG resistance by 10-fold compared with lower-level resistance associated with R263K mutation alone. However, to date, the double-mutant E157Q-R263K has not yet been described in vivo at VF of an INI-based regimen [18].

Interestingly, regarding both INI currently under development, BIC and CAB, they remained active against R263K-M50I and R263K-E138K double-mutants with less than ≤4-fold increase in phenotypic resistance level [7].

### 2.6. R263K and HIV Viral Subtype

The R263K substitution was reported at VF in a subtype C-infected participant in the SAILING clinical trial [1]. Thus, in vitro characteristics of a R263K site-directed mutant in the subtype C context was assessed showing a significant decrease in strand-transfer activity for the R263K integrase protein to a greater extent than observed in subtype B [19]. The presence of R263K decreased HIV-1 subtype C infectiousness by 70% compared to wild-type, whereas R263K in subtype B only resulted in a 40% decrease [19]. Thus, the R263K substitution appears to be more deleterious in subtype C than in subtype B.

### 2.7. Selection of R263K Mutation and What Behind: In Vitro Data

In the study of Anstett et al., tissue culture selections with DTG, using viruses resistant to first-generation INIs (RAL and EVG), infectivity and resistance assays were performed. They showed that the presence of the E92Q or N155H resistance mutations was compatible with the emergence of R263K, whereas no R263K selection was observed in presence of G140S-Q148R, E92Q-N155H, G140S, Y143R and Q148R resistance mutations [20]. Thus, some genotypic resistance profiles seem to prevent emergence of the R263K DTG resistance-associated mutation.

In the opposite way, in vitro the presence of the R263K delayed the emergence of RAL resistance-associated mutations, whereas the simultaneous presence of either the H51Y or E138K substitutions in combination with R263K somewhat mitigated this inhibitory effect. In contrast, in vitro experiments showed that, in the presence of R263K mutation, resistance selection to EVG appeared earlier than in wild-type virus [21].

The N155H is one of the major resistance pathway to the first-generation INI, and is often associated with secondary resistance mutations. In the study of Anstett et al., L74M, E92Q, T97A, E157Q and G163R resistance mutations were introduced into NL4.3 subtype B HIV-1 vectors harboring N155H and R263K in tandem [22]. They found that the addition of T97A, E157Q or G163R mutation somewhat improved the affinity of the double-mutant N155H-R263K for its target DNA substrate, while the presence of L74M or E92Q had a negative effect on this step. This work showed that the compensatory mutations, that evolve after N155H selection under continued DTG or RAL/EVG pressure, are unable to improve enzyme efficiency, resistance level or viral infectivity in an N155H-R263K background [22].

The study of Singhroy et al. assessed the impact of adding the M184I/V mutation to a R263K-mutated virus on viral replicative capacity. Their results showed that the presence of M184I or M184V with R263K further decreased viral infectiousness and replicative capacity compared to the effects of the individual mutations alone [23].

## 3. E157Q Integrase Mutation

### 3.1. Prevalence of E157Q in cART-Naïve Patients

Integrase E157Q substitution has been described as a polymorphism present in 2.4% of viral sequences obtained from cART-naïve patients in the ARCA Italian database and in 5.0% in the last French transmitted drug resistance survey study [3,24]. A recent study of the French ANRS AC11 virology network conducted on 8528 integrase sequences from INI-naïve patients showed that the overall prevalence of E157Q polymorphism was 2.7% and its distribution among HIV-1 subtypes was 1.7%, 5.6% and 2.2% in B, CRF02_AG and others non-B subtypes, respectively [25].

### 3.2. In Vivo Selection of E157Q Mutation at Virological Failure

E157Q emergence in integrase has also been observed in the case of VF under a RAL-based treatment in two case reports. A case report in an highly cART-experienced patient has shown that mutation E157Q was rapidly selected and archived in intracellular HIV DNA within a short term of eight weeks of low-level replication under a RAL-based treatment [4]. Indeed, in this case report mutation at position 157 of integrase was not present in baseline plasma virus [4]. In a second study based on nine patients experiencing a VF under a RAL-based treatment, E157Q mutation was one of the four different resistance mutations profiles identified at VF [5].

### 3.3. Phenotypic Analysis of E157Q Mutants

A recent Italian study carried out in vitro phenotypic assays on six clinical samples harbouring E157Q-mutated viruses including three subtype B and three CRF02_AG recombinant, showing they were all susceptible to DTG and to RAL, except one at the limit of the biological cut-off for RAL [3]. The study of Shimura et al. assessed susceptibility to EVG of a E157Q recombinant molecular clone showing a small reduction in EVG susceptibility with a FC of 6.3 [26].

These assays were mainly conducted with a subtype B. In a recent study, we carried out phenotypic assays using E157Q mutant generated in the subtype B context, but also in the CRF02_AG context. In this study, the E157Q site-directed mutants did not show an increased phenotypic resistance level to DTG or RAL both in B and CRF02_AG subtypes contexts [24]. These in vitro phenotypic data are in accordance with the recent findings of the Italian study using similar phenotypic assays [3]. However, we observed a slight increase of FC to EVG at 1.9 and 2.4 in the presence of E157Q in B and CRF02_AG contexts, respectively [25]. Thus, E157Q polymorphism does not seem to impact phenotypic susceptibility to RAL or DTG, in contrast a potential low-level resistance, especially in the context of CRF02_AG recombinant, was observed for EVG.

### 3.4. Virological Response to an INI-Based Regimen

A case report of a non virological response to a DTG-based regimen, in a patient infected with a E157Q-mutated virus, has been described despite adequate DTG plasma concentrations [6]. In vitro characterization of the E157Q-mutated virus issued from this clinical sample has shown that integrase strand-transfer activity was 3-fold greater compared to wild-type virus and that IC_50_ value was increased, leading to a FC equal to 9 [6].

To date, very few data are available regarding virological response of patients displaying E157Q-mutated virus, only represented by case reports that limit their interpretation. In the study of Ambrosioni et al., only two cART-naïve patients initiated a DTG-based regimen with a very short follow-up of 12 weeks [27]. In the study of Pavkovich et al., only six among the 15 patients initiating an INI-based regimen were in virological success at month 12; however heterogeneous profiles of patients, cART-naïve and cART-experienced, were mixed in the analysis [28].

A recent study of the French ANRS AC11 virology network assessed the virological outcome of 39 INI-naïve patients with E157Q-mutated virus initiating an INI-based regimen [25]. Among them, 15 had a viral load (VL) < 50 c/mL at initiation and virological suppression was maintained during the first-year follow-up in all but two exhibiting a viral blip. Twenty-four patients had a VL > 50 c/mL at time of INI-based regimen initiation. Among them eight were receiving their first-line c ART and the two patients who did not reach VL < 50 c/mL at W24 were receiving EVG-based single-tablet regimen. The 16 remaining patients were cART-experienced in VF with drug-resistant viruses and they displayed several virological outcomes to the INI-based regimen, independently of the genotypic susceptibility score of the cART. Thus, taking into account all the data, in case of E157Q polymorphism, the most recommended INI might be DTG in such patients; EVG should not be considered due to potential low-level resistance, especially in the context of CRF02_AG recombinant and RAL seems adequate with unmounted IC_50_ but with the caveat that selection of E157Q has been described at VF in two case reports.

## 4. Conclusions

These findings show that there is still a need for better understanding of resistance mechanisms to INI, specially for DTG. These findings also emphasized the importance of genotypic background in viral evolution under drug pressure and that high level of resistance could result from combined integrase polymorphisms as yet unidentified.
